# A Novel BN Learning Algorithm Based on Block Learning Strategy

**DOI:** 10.3390/s20216357

**Published:** 2020-11-07

**Authors:** Xinyu Li, Xiaoguang Gao, Chenfeng Wang

**Affiliations:** School of Electronic and Information, Northwestern Polytechnical University, Xi’an 710129, China; lxy_2017@mail.nwpu.edu.cn (X.L.); chen-cc@mail.nwpu.edu.cn (C.W.)

**Keywords:** Bayesian networks, block learning, high-dimensional and sparse data, structure learning

## Abstract

Learning accurate Bayesian Network (BN) structures of high-dimensional and sparse data is difficult because of high computation complexity. To learn the accurate structure for high-dimensional and sparse data faster, this paper adopts a divide and conquer strategy and proposes a block learning algorithm with a mutual information based K-means algorithm (BLMKM algorithm). This method utilizes an improved K-means algorithm to block the nodes in BN and a maximum minimum parents and children (MMPC) algorithm to obtain the whole skeleton of BN and find possible graph structures based on separated blocks. Then, a pruned dynamic programming algorithm is performed sequentially for all possible graph structures to get possible BNs and find the best BN by scoring function. Experiments show that for high-dimensional and sparse data, the BLMKM algorithm can achieve the same accuracy in a reasonable time compared with non-blocking classical learning algorithms. Compared to the existing block learning algorithms, the BLMKM algorithm has a time advantage on the basis of ensuring accuracy. The analysis of the real radar effect mechanism dataset proves that BLMKM algorithm can quickly establish a global and accurate causality model to find the cause of interference, predict the detecting result, and guide the parameters optimization. BLMKM algorithm is efficient for BN learning and has practical application value.

## 1. Introduction

With the rapid development of machine learning theory, Bayesian Networks (BN) as a powerful causal representation model were proposed by Judea Pearl in 1986 [1], which can systematically describe the causality between random variables. BN consists of a directed acyclic graph (DAG) and conditional probability tables (CPTs). The directed acyclic graph can intuitively and qualitatively represent the conditional independence relationships between variables; the conditional probability tables quantitatively represent the probability dependence relationships between variables. Simultaneously, BN has a strict mathematical foundation and an intuitive network topology, its natural causal analysis of real problems makes the entire model construction and decision-making process extremely interpretable. BN is good at solving uncertain modeling and reasoning of complex systems, often used for practical problems of “causal traceability,” such as genetic analysis [2,3], medical diagnosis [4,5,6], reliability analysis [7], fault detection [8], pattern classification [9] and other fields.

The first step of BN learning is to construct DAG and then learn the CPTs of all variables. This process corresponds to structure learning [10] and parameter learning [11] in turn. Learning a BN structure is the basis of learning parameters and the key to the whole BN learning. Based on the constructed structure and parameters, the inference is carried out to complete the process of causal traceability. Therefore, the learning accuracy and speed of the BN structure play an important role.

There are different BN structure learning algorithms for different data dimensions. Normally, when learning low-dimensional data, exact learning algorithms are widely used to get the optimal BN model because of their high accuracy; approximate learning algorithms are also available. When the data dimension increases, the dimensional explosion of accurate learning algorithms leads to the time complexity increasing sharply. When the number of variables is greater than 26, the results can no longer be obtained within a reasonable time [12]; exact learning algorithms are limited for higher-dimensional data. If we do not have a high requirement for accuracy of BN structure, approximate learning algorithms can learn the BN structure in a shorter time; another idea is that, because of the sparse characteristics of high-dimensional data, BN structure learning can be partitioned into several stages and carried out independently in turn. Finally, the entire BN structure learning can be completed through integration. This is the strategy of novel block structure learning algorithms.

As mentioned above, for this kind of high-dimensional and sparse data, BN structure learning algorithms are currently divided into two types: non-blocking classical structure learning algorithms and novel block structure learning algorithms (both are approximate learning). The non-blocking classical structure learning algorithms used in high-dimensional and sparse data directly learn BN structure from the data. These approximate learning algorithms are feasible, and the complexity is controlled in a reasonable range for smaller scales of BNs. Think about performing block processing before network learning. At present, there are some research results on the block structure learning algorithm. Koller and Friedman demonstrated the advantages and necessity of using sparse models to improve the generalization ability of network models [13]. Li Shuohao et al. proposed a graph segmentation method in 2014 [14] which can effectively reduce the time consumption of large-scale BN learning. This method uses the hill-climbing algorithm or greedy search algorithm to learn the structure of the graph after segmentation; these two algorithms are learning algorithms based on scoring function, which have inherent weaknesses and are easy to fall into local optimum. The SAR algorithm [15] proposed by Hui Liu et al. in 2017 pointed out that the task of learning large DAGs can be decomposed into relatively small-scale DAGs using a q-partial graph and the strategy of minimum node separation and reunifying these small DAGs builds the entire DAG. This algorithm is currently the latest block learning algorithm, but it uses a learning algorithm based on a scoring function to learn small DAGs, does not use constraints to reduce the search space, and has inherent weaknesses of approximate learning.

The above algorithms are designed to solve the BN structural learning of high-dimensional data when the number of nodes increases, but because the learning algorithm still uses approximate learning algorithms, there is no guarantee that the final obtained model will be optimal. At this point, it may be a local optimal network or the accuracy may be limited. Although large-scale BN block approximation learning has made some achievements, there have been no related reports on the study of block exact learning.

Indeed, after a BN is obtained, we usually use it to infer some unknown variables with several variables known. From this point of view, the complexity of the inference algorithm can reflect the complexity of data. Take the junction tree algorithm [16] adopted in this paper as an example, the complexity is exponential in the treewidth, which is related to the number of variables in the data. Hence, the complexity of data is also exponential in the number of variables. As the scale of the network becomes larger, exact learning will face more challenges. For example, how to ensure that the speed of the algorithm is improved while maintaining the accuracy; how to avoid finding a local optimal network when searching for a complex candidate space so that the resulting network must be globally optimal; how to improve accuracy without increasing time consumption, etc.

This paper adopts the strategy of separation and combination and proposes a novel block learning algorithm to reduce the complexity of BN structure learning for high-dimensional and sparse data. The proposed algorithm improves the speed of the algorithm while ensuring the accuracy of the acquired network, and also ensures that the network found is globally optimal, which is also applicable to such problems as the analysis of radar effect mechanism.

First, we must find a block algorithm suitable for BN. This paper analyzes the existing block algorithm (that is, clustering algorithm, hereinafter referred to as the block algorithm), and finds the classic prototype clustering algorithm, the K-means algorithm [17], is intuitive and clear in describing the process of partitioning. Therefore, the K-means algorithm is used to partition the network in this paper. It is efficient and fast to judge whether the nodes in BN are clustered into a block by distance, and it can express the clustering process well. However, when measuring the distance between BN nodes, the Euclidean distance metric used by the original K-means algorithm represents the dissimilarity of the sample state, which is inconsistent with the problem we studied, so we need to improve the K-means algorithm. Mutual information is based on the probability value of the samples and is often used to measure the correlation of each node of BN. Therefore, this paper proposes a mutual information based K-means algorithm (MKM algorithm) to partition the network. MKM algorithm is used to effectively partition the network to obtain the network’s block structure. Secondly, according to the architecture of the whole network, all related edges between blocks are found, and all possible graph structures are found by assuming the possible connection directions of all edges between blocks. Next, the dynamic programming algorithm is used to learn the structures of all possible graph structures. Finally, find the optimal BN according to the scoring function. Based on this, a block learning algorithm (BLMKM algorithm) is proposed in this paper.

Different from the existing block learning algorithms, the BLMKM algorithm uses a MKM algorithm to obtain the better block structure of the network, and the pruned dynamic programming algorithm is used in the structure learning part to realize exact learning of the network. Through the proposed BLMKM algorithm, the BN model can be constructed more quickly, and the actual characteristics of high-dimensional and sparse data can be further described more efficiently. Meanwhile, the BLMKM algorithm can be used to construct a BN model for high-dimensional and sparse data in the real world, for example, the analysis of the radar effect mechanism. By studying the influence of various factors on the radar detection accuracy, we can complete tasks such as radar detection error analysis; that is, the analysis of radar effect mechanism. At present, the radar effect mechanism analysis mainly adopts the traditional equation calculation method, which is complicated, slow, and unable to infer backward. BN is an advantageous tool for causal representation and analysis and can be introduced to solve this problem [18,19]. Because there are many variables affecting radar detection and the radar system is composed of multiple subsystems, the data of radar effect mechanism are high-dimensional and sparse. The proposed BLMKM algorithm can model this high-dimensional and sparse data efficiently, and the inference is based on the model. The BLMKM algorithm proposed in this paper is a new and effective way to analyze the mechanism of radar effect.

The organizational structure of this paper is as follows: Section 2 briefly introduces the relevant theories of BN structure learning and network block; Section 3 details the proposed BLMKM algorithm and prediction method, including the idea of a BLMKM algorithm and the related algorithms involved in a BLMKM algorithm; Section 4 performs simulation experiments to verify the performance of BLMKM algorithm and uses BLMKM algorithm for analysis of radar effect mechanism. Section 5 briefly summarizes the work of this paper.

## 2. Related Theory

### 2.1. BN and Structure Learning

BN is a directed acyclic graph (DAG) with conditional probability tables (CPTs). Nodes and edges in DAG describe conditional dependencies, and CPTs describe the distribution of the variables. Hence, BN can be defined as BN=(G,P). One node in DAG can parameterized by P(node|pa(node)), where pa(node) represents parent nodes (if any).

In BN, the corresponding full probability formula is:

(1) $$P(X_{1},X_{2},\ldots ,X_{n})=P(X_{1})P(X_{2}|X_{1})\ldots P(X_{n}|X_{1},X_{2},\ldots ,X_{n-1})$$

$X_{1},X_{2},\ldots ,X_{n}$ represents variables.

BN structure learning is to determine the structure G and parameters P of BN at the same time through data analysis without knowing the network structure G. The dynamic programming algorithm [20] divides the BN learning process into two stages: finding the parent node graph parentGraph and finding the node sequence graph orderGraph. For node $X_{i}$, $parentGraph(X_{i})$ is a directed graph composed of $X_{i}$ and possible parent nodes $pa(X_{i})$, here $pa(X_{i})$ is the power set formed by all nodes except for $X_{i}$; orderGraph is the power set formed by all nodes in the network in a certain order. The dynamic programming algorithm starts with a zero-node subnet, iterates and evaluates parentGraph and orderGraph in turn, and adds nodes recursively to the subnet until all nodes are added to the network. The evaluation criterion is the MDL scoring function:

(2) $$MDL(V)=\underset{X\in V}{\min}\left\{MDL\left(V\backslash \left\{X\right\}\right)+BestMDL\left(X,V\backslash \left\{X\right\}\right)\right\}$$

(3) $$BestMDL\left(X,V\backslash \left\{X\right\}\right)=\underset{pa(X)\subseteq V\backslash \{X\}}{\min}MDL(X|pa(X))$$

For the practical problem with a slightly larger number of nodes, the number of parent nodes pa(X) may increase exponentially. The dimensional disaster is a major disadvantage of the dynamic programming algorithm.

### 2.2. Blocking Algorithms

Blocking the network is to find the community structure of the network. The community structure means that the nodes in the network can be partitioned into clusters, the connections between the nodes in one cluster are dense, and the connections between clusters are sparse. Clustering is an unsupervised learning process that partitions data into meaningful or useful clusters based on similarity. Therefore, the problem of finding community structure is essentially a clustering problem, which is what we call the blocking problem.

There are three main types of blocking algorithms. The first type of algorithm assumes that the blocking structure can be characterized by the prototype, called prototype-based blocking algorithm. With the assumption of prototype, this type of algorithm is usually used for real-world classification tasks. The most famous one of the prototype-based blocking algorithms is the K-means algorithm. On this basis, this paper proposes the MKM algorithm to partition BN into blocks. The second type is the density-based blocking algorithm. Different from prototype-based blocking algorithm, it blocks the data based on the density in the spatial distribution and determines the blocking structure by the compactness between samples. DBSCAN [21,22] is a well-known density-based blocking algorithm which describes the tightness of the sample’s distribution based on domain parameters and can partition arbitrarily shaped blocks from a noisy spatial dataset. The third type is a hierarchical blocking algorithm, such as the agglomeration algorithm and splitting algorithm. FastNewman algorithm (FN algorithm) [23] is a typical agglomeration algorithm mainly used in weighted networks. It adapts greedy thoughts to merge communities from bottom to top according to the criteria for maximizing the degree of modularity. This algorithm is of great significance for the research of practical networks. Therefore, we will carry out a comparison experiment between the proposed MKM algorithm and FN algorithm.

## 3. BLMKM Algorithm

### 3.1. MKM Algorithm

Before learning large-scale sparse BN structure, the nodes in the network should be firstly partitioned. The K-means algorithm is simple and transferable, and it is more efficient than other block algorithms. Therefore, in this paper, a mutual information based K-means algorithm (MKM algorithm) is proposed based on the K-means algorithm.

The conventional K-means algorithm is unreasonable when blocking BN. On the one hand, the K-means algorithm uses Euclidean distance to identify the similarity between the nodes. However, for two nodes in BN, the Euclidean distance measures the similarity between states, which cannot correspond to the physical meaning of the problem we are studying. On the other hand, during the training of the conventional K-means algorithm, the mean of all data samples in each cluster is used as the new cluster center. But this cluster center may not exist in the BN, and it has no practical significance for other nodes to merge to the community of this virtual point.

Given this two unreasonableness, this paper improves the K-means algorithm and proposes an improved K-means algorithm (MKM algorithm) to adapt it to the block task of BN.

Mutual information [24] is used as the measure of the dependency relationship between nodes.

Mutual information is the entanglement of two pieces of information in a joint distribution, that is

(4) $$I(X;Y)=\sum_{x\in X}\sum_{y\in Y}p(x,y)\log\frac{p(x,y)}{p(x)p(y)}$$

This function expresses the intrinsic dependence between the joint distribution of X and Y relative to the joint distribution under the assumption that X and Y are independent. Hence, mutual information can be used as an effective measure of the relationship between different variables, as well as the nodes correlation in BN. If mutual information is used as a distance measure to replace Euclidean distance, it must satisfy the following basic properties:

(1) Non-negative: $d(X_{i},X_{j})\geq 0$;
(2) Identity: $d(X_{i},X_{j})=0$ if and only if $X_{i}=X_{j}$;
(3) Symmetry: $d(X_{i},X_{j})=d(X_{j},X_{i})$;
(4) Directivity: $d(X_{i},X_{j})\leq d(X_{i},X_{k})+d(X_{k},X_{j})$.

Actually, mutual information satisfies all these properties:

(a) Non-negative: Since the mutual information is from the overall perspective of the random variables *X* and *Y*, and the problem is observed in the average sense, the average mutual information amount will not appear negative, I(X;Y)≥0.
(b) Identity: I(X;Y)=0 if and only if *X* and *Y* are independent random variables. When *X* and *Y* are independent, p(x,y)=p(x)p(y), therefore $\log\frac{p(x,y)}{p(x)p(y)}=\log1=0$.
(c) Symmetry: I(X;Y)=I(Y;X), I(X;Y) and I(Y;X) just stand on different ground, the amount of information about *X* extracted by *Y* is the same as the amount of information about *Y* extracted from *X*. From another perspective, I(X;Y)=H(X)+H(Y)−H(X,Y)=I(Y;X).
(d) Directivity: I(X;Y)≤I(X;Z)+I(Z;Y), if a Markov chain X→Z→Y is formed, the average mutual information between the input message and the output message tends to become smaller as the number of processors increases after the message is processed at multiple levels, that is, I(X;Y)≤I(X;Z), I(X;Y)≤I(X;Z).

It can be seen that mutual information can measure the dependency relationship between nodes in BN. Using mutual information as the metric, we can effectively block the network with practical significance based on the correlation between nodes, while the complexity of MKM is O(n⋅k⋅l), in which n, k, l represent the number of variables, blocks and iterations, respectively.

As we have seen, the new center node generated by the K-means algorithm through constant iterations is probably not a real node in the cluster. If some anomalous nodes are relatively far from the center node, the recalculated center node may likely deviate from the true center of the cluster. For Bayesian Networks, the new center node will be virtual, which is not applicable. Therefore, this paper draws on the idea of the K-center node algorithm, and repeatedly replaces central nodes with non-central nodes, trying to find a better center node to improve the quality of clustering. This idea can reduce the influence of anomalous nodes on clustering.

This paper uses the cost function of mutual information to measure the quality of clustering, and the total cost is the sum of the mutual information of all nodes in the subset. During iteration, if the value of the cost function of the new subset is greater than the value of the cost function of the original subset, the new center node is used to replace the old center node until the center does not change.

**Figure 1:**
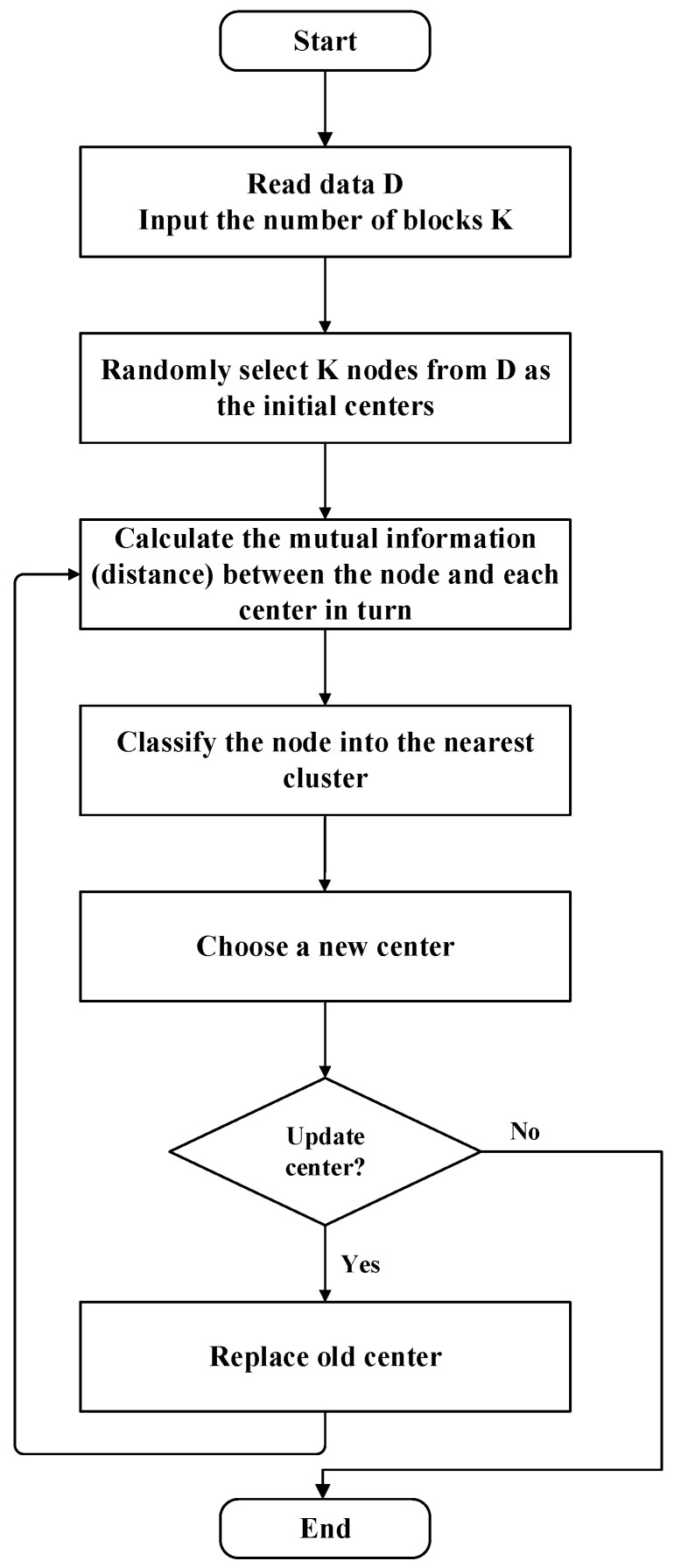
Flowchart of mutual information based K-means algorithm (MKM algorithm).

According to the above introduction, the flowchart of the mutual information based K-means algorithm (MKM algorithm) is as Figure 1.

### 3.2. BLMKM Algorithm

After finding blocks by the MKM algorithm, consider the sparseness of large-scale networks: variable connections between blocks are sparse, and variable connections within blocks are tight. Therefore, after the BN structure in these blocks is learned, we can first find the undirected edges between blocks through the network skeleton, then assume the direction of the edges between the blocks to obtain the possible graph structure between all blocks, and finally perform exact learning in turn to get optimal BN. The above is the research idea of the BLMKM algorithm, which can reduce the complexity of learning large-scale networks.

This paper references the MMPC algorithm [25] to obtain the undirected graph skeleton of the network. The MMPC algorithm finds each node’s neighbors (the set of parents and children, that is, PC) from the input data and approximates the network structure. As for the node $X_{i}$, the potential neighbor nodes are other nodes except the node $X_{i}$, and according to the maximum and minimum heuristic method, select node $X_{j}$ that maximizes the smallest connection with $X_{i}$ and add it to PC of node $X_{i}$. After getting the preliminary PC, given the node S in PC, if the node $X_{i}$ and the node Y in PC are independent, then remove Y (prove that $X_{i}$ and Y are not a parent–child relationship). The MMPC algorithm finally returns a 0–1 matrix to represent the network skeleton. We define the network skeleton as MB. For larger networks, multiple independence tests may take a lot of time to find a more accurate architecture. The complexity of MMPC is $O(n\cdot PC^{m})$ [26], n means the number of variables, PC represent the set of parents and children sets for all the nodes, and m means the number of internal iterations in MMPC.

**Figure 2:**
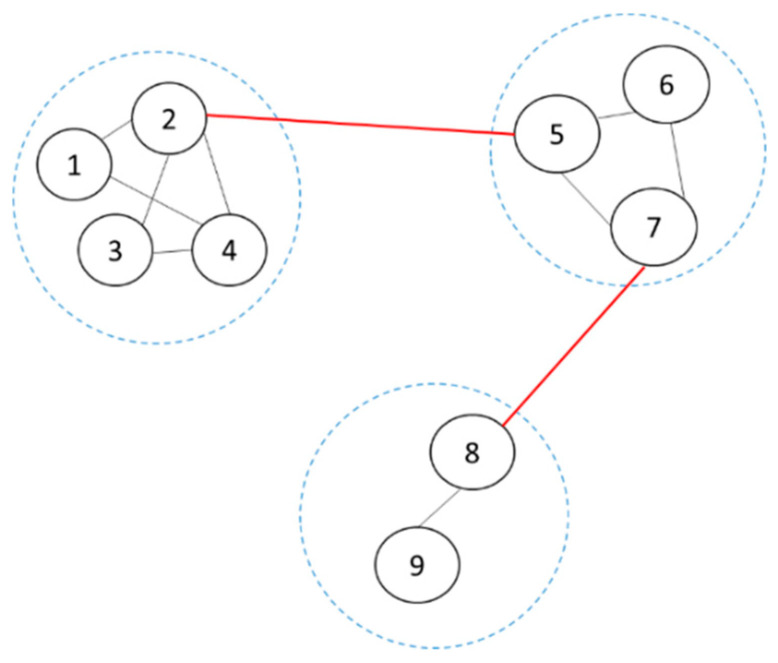
Schematic diagram of the network structure before combining.

After using the MKM algorithm and MMPC algorithm to obtain blocks and the network skeleton, respectively, the schematic diagram of the network at this point is shown in Figure 2.

This undirected structure is the network skeleton obtained by the MMPC algorithm. Three blue virtual circles represent the blocks obtained by the MKM algorithm: each block is an undirected network, there are more connecting edges (black edges) in the block, and the connections within the block are tight. The connections between three blocks (red edges) are also determined by the network skeleton, and the connections between the blocks are sparse.

Next, the direction of the undirected edges between the blocks are assumed to find the possible graph structures among all the blocks. Assume that there are n undirected edges (red edges), there may be $2^{n}$ combinations of the directions of the edge arrows, and there may be $2^{n}$ possible graph structures among all the blocks. Store the possible graph structures in turn for later structure learning. This is what we called the combine function.

**Figure 3:**
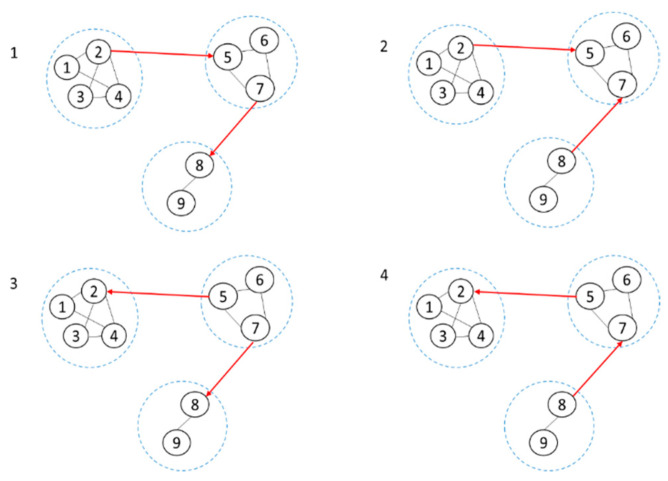
Four possible graph structures among all the blocks corresponding to Figure 2.

In Figure 2, there are two red undirected edges among all the blocks leading to four possible graph structures, which, respectively, are (2→5,7→8),(2→5,8→7),(5→2,7→8), (5→2,8→7) in Figure 3.

We need to learn all the possible graph structures in Figure 3 in sequence to select the best BN by score function. This strategy ensures that the resulting network is globally optimal because the candidate BNs obtained after the structural learning of each possible graph structure are globally optimal, therefore, the best BN can be selected by score function. As shown in Figure 3, for the first possible graph structure (2→5,7→8), when performing structural learning on the block composed of nodes 5, 6, and 7, the score of node 5 needs to consider the score of node 2 as the parent node. Similarly, when performing structural learning on the block composed of nodes 8 and 9, the score of node 8 needs to consider the score of node 7 as the parent node. This ensures that other possible parent nodes outside the block are not missed after blocking. The calculation of the network score is considered globally. At this time, the structure learned is globally optimal, and the network finally found is also globally optimal.

Dynamic programming algorithms are used for structural learning. This algorithm is a kind of exact learning algorithm which divides the BN structure learning process into two steps: finding parent graphs and order graphs, and recursively solving the problem using the MDL score to measure the matching degree of candidate structures and data.

The MDL score considers the compromise between data matching and network complexity. The state’s number of node $X_{i}$ is $r_{i}$, the occurrence number of $PA_{i}=pa_{i}$ is $N_{pa_{i}}$, $N_{X_{i},pa_{i}}$ represents the number of simultaneous occurrences of $PA_{i}=pa_{i}$ and $X_{i}{=x}_{i}$. The first term in Equation (5) is based on entropy, and the second term is a penalty term for network complexity. Then the MDL score of a structure can be expressed as follows [27]:

(5) $$MDL(G)=\sum_{i}MDL(X_{i}|PA_{i})$$

(6) $$MDL(X_{i}|PA_{i})=H(X_{i}|PA_{i})+\frac{\log N}{2}K(X_{i}|PA_{i})$$

(7) $$H(X_{i}|PA_{i})=-\sum_{x_{i},pa_{i}}N_{x_{i},pa_{i}}\log\frac{N_{x_{i},pa_{i}}}{N_{pa_{i}}}$$

(8) $$K(X_{i}|PA_{i})=(r_{i}-1)\prod_{X_{l}\in PA_{i}}r_{l}$$

The MDL score is decomposable [28], that is, the score of the entire structure can be obtained by summing each variable.

The dynamic programming algorithm starts with an empty network, and then gradually adds leaf nodes recursively until all variables are added to the network, and finally finds the optimal network.

**Figure 4:**
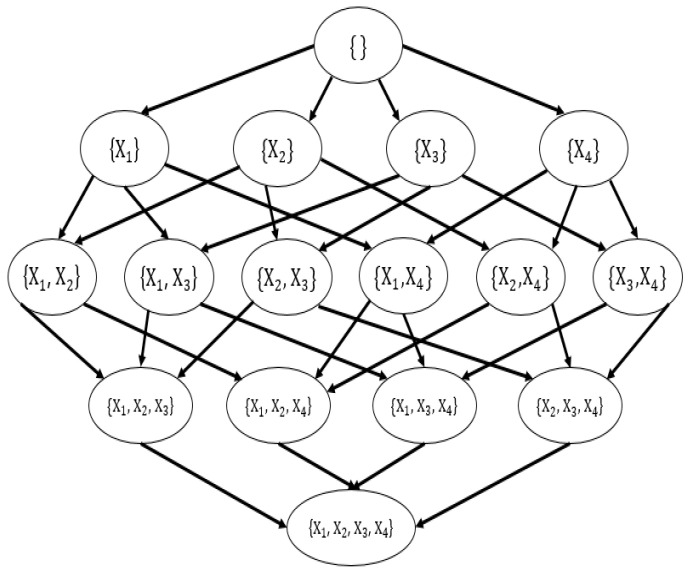
The order graph of four variables.

This paper uses order graphs and parent graphs to show how the dynamic programming algorithm recursively decomposes to find the optimal network. Figure 4 shows the order graph of four variables. The order graph of n nodes has $2^{n}$ subsets. All nodes in the l layer have l precursor nodes in the previous layer and layer l has C(n,l) nodes, where C(n,k) is the binomial coefficient.

**Figure 5:**
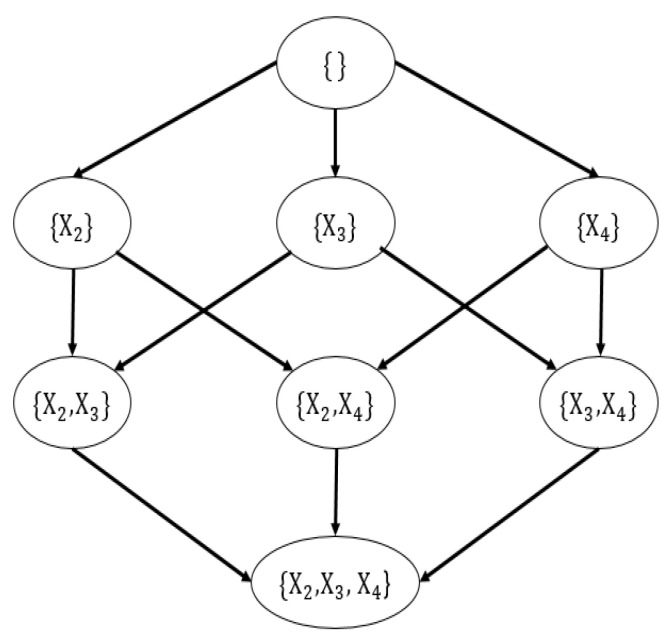
The parent graph of node $X_{1}$.

In the order graph, each node selects the precursor nodes that make the subnetwork optimal based on the MDL score. Therefore, when evaluating node O in the order graph, take each X∈V\O as a leaf node, take O to be a subnetwork, and the score of the new subnetwork is MDL(O∪X)=MDL(O)+BestMDL(X,O). The parent set and the score of the optimal leaf node will be kept in the subnetwork. After considering the precursor nodes of all nodes in the order graph, the optimal subnetwork of the corresponding variable set is included. The path from the root node to a single leaf node represents the order in which nodes are added to the network, so it is called the order graph. This order partly determines the network structure, and the rest of the structure is determined by the parent set of each node. To find the optimal parent set for each node, use the parent graph to calculate BestMDL(X,U). Figure 5 shows the parent graph of node $X_{1}$.

Each node corresponds to a parent graph. Figure 5 contains a subset of n−1 other nodes, with a total of $2^{n-1}$ variables. Each variable in the parent graph represents a mapping from a candidate parent set to a subset of these variables; this mapping is the one that optimizes the $X_{i}$ score. All nodes of the same layer have the same number of possible parent nodes. Besides, all nodes in the l layer have l precursor nodes in the previous layer, and the l layer has C(n−1,1) nodes. To evaluate the nodes in the candidate parent set P of node X, for each Y∈P, consider BestMDL(X,P\{Y}) and MDL(X|P). The best-scoring node is stored as BestMDL(X,P). By P storing the optimal score of each candidate parent set, after considering all the precursor nodes, it includes all the subsets of variable X and candidate parent set with the optimal score.

**Figure 6:**
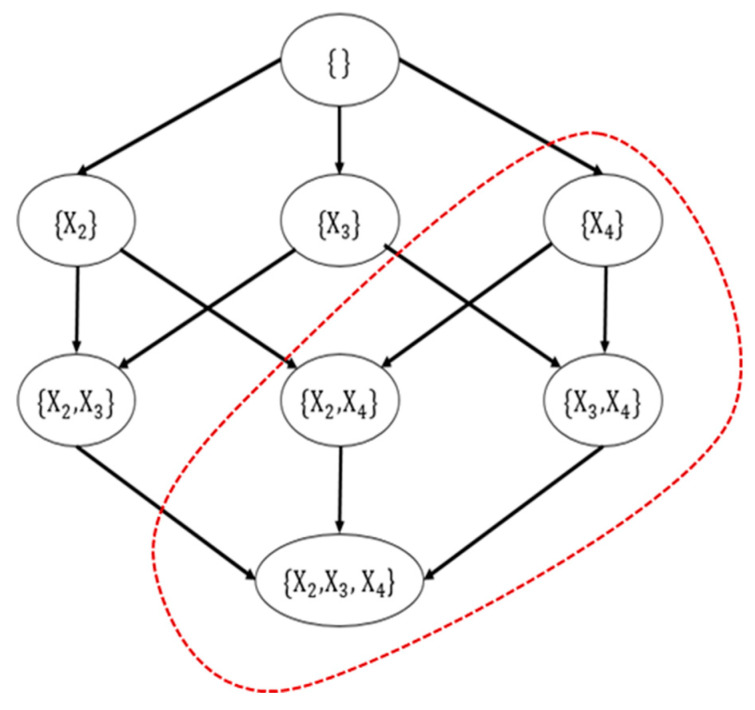
Pruned parent graph of node $X_{1}$.

In this paper, we use the network skeleton MB obtained by the MMPC to prune Figure 5. Take Figure 6 as an example, assume that the neighbor nodes of $X_{1}$ obtained by the MMPC algorithm are only $X_{2}$ and $X_{3}$, which means $MB_{X_{1}}=\{X_{2},X_{3}\}$. At this time, the parent graph of $X_{1}$ should prune all sets containing $X_{4}$. Similarly, P in line 16 of Algorithm 1 will be selected from MB, instead of all other nodes except P. So we can use MB to prune the structure. The original complexity of dynamic programming is $O(n\cdot 2^{n-1})$, with MKM to block the whole network and the network skeleton MB obtained by MMPC to prune the structure, n can be reduced to a low level. Even the complexity may degenerate from exponential to polynomial. These methods can significantly reduce time complexity compared to other none-blocking structure learning algorithms.

The flow of the pruned dynamic programming algorithm is shown as Algorithm 1, which refers to the original dynamic programming in [20] and improves the pruned strategy of it.
**Algorithm 1** Pruned Dynamic Programming Algorithm**procedure**GenerateLayer(orderGraph)1. **for** each parentGraph
pg
**do**2. generateParentLayer(pg)
3. **end for**4. **for** each node O∈prev
**do**5. **for** each v∈V−O
**do**6. pg←parentGraphs[v].readNode()
7. score←pg.score+O.score
8. **if**
score<curr[O∪v].score
**then**9. curr[O∪v]←{O.pars∪pg.pars,score}
10. **end if**11. **if**
$v=Y_{1}$
**then**
write(curr[O∪v])
12. **end for**
[each
v]
13. **end for**
[each
node
O]
14. prev←curr
15. curr←newHashTable
**end procedure**
**procedure**GenerateParentLayer(pg)16. **for** each node P∈MB
**do**17. **for** each v∈V−P and v≠pg.v
**do**18. **if**
curr[P∪v] is null **then**19. curr[P∪v]←{P∪v,score(P∪v)}
20. **end if**21. **if**
P.score<curr[P∪v].score
**then**22. curr[P∪v]←P
23. **end if**24. **if**
$v=p_{1}$
**then**
write(curr[P∪v])
25. **end for**
[each
v]
26. **end for**
[each
node
P]
27. prev←currentLayer
28. curr←newHashTable
**end procedure**
**procedure***main*29. **for**
l=1→n
**do**30. GenerateLayer(orderGraph)
31. **end for****end procedure**

In summary, this paper proposes the BLMKM algorithm for BN modeling of high-dimensional data, which can be summarized as the following steps: In the first step, the data is blocked by the proposed MKM algorithm; in the second step, the MMPC algorithm is used to determine the network skeleton; in the third step, the combine function assumes the direction of the edges between the blocks to find the possible graph structures between all the blocks. In the fourth step, the pruned dynamic programming learning is performed on the possible graph structures between all the blocks. Finally, the optimal network is found using the BIC score. The BLMKM algorithm is as Algorithm 2.
**Algorithm 2** BLMKM Algorithm**Input:** dataset D, number of clusters k**Output:** optimal BN $N_{G}^{*}$ and score $s^{*}$
1. $C=\{C_{1},C_{2},\ldots ,C_{k}\}\leftarrow \mathrm{MKM}(D,k)$
2. The skeleton of the network (MB)←MMPC(D)
3. $All\;possible\;diagrams\;\delta^{*}\leftarrow \mathrm{Combine}(C,The\;skeleton\;of\;the\;network)$
4. **for**
$i=1,2,\ldots 2^{m}$
**do**5. $N_{G_{i}}^{*}\;and\;s_{i}{}^{*}\leftarrow \mathrm{Pruned}\;\mathrm{Dynamic}\;\mathrm{Programming}(D,\delta_{i}^{*})$
6. **end for**7. Optimal BN $N_{G}^{*}\leftarrow \arg\max(s_{i}{}^{*},N_{G_{i}}^{*})\;\mathrm{and}\;s^{*}\leftarrow \max(s_{i}{}^{*},N_{G_{i}}^{*})$


The BLMKM algorithm will slightly increase the learning time of the entire network due to learning the skeleton, but before the learning block the network greatly reduces the space consumption and improves the efficiency of the algorithm. Especially for the dynamic programming algorithm, the divide and conquer strategy significantly reduces the size of the network needed to learn, while MMPC helps to prune the parent graph to avoid unnecessary calculation. The BLMKM algorithm proposed in this paper can improve the running speed while ensuring accuracy for BN modeling of high-dimensional data.

## 4. Experiments

### 4.1. Experiments of BLMKM Algorithm on Classic Networks

We used the three classic networks in the BN repository library and the networks Mybnet1 and Mybnet2 to verify the performance of the BLMKM algorithm. The parameters of the networks are as Table 1:

#### 4.1.1. Comparison of Algorithm’s Running Time

**Figure 7:**
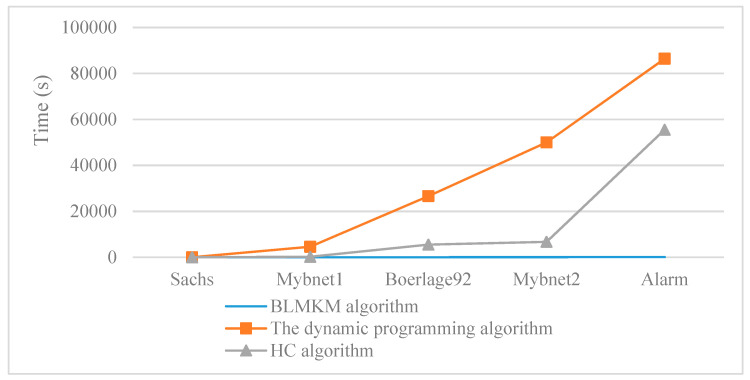
The comparison of running time between a BLMKM algorithm and other non-blocking classical structure learning algorithms.

We compared the running time of BLMKM algorithm with non-blocking classical structure learning algorithms, as shown in Figure 7. The dynamic programming algorithm [20] is a conventional exact learning algorithm, and the HC algorithm [24] is a classic learning algorithm based on a scoring function. This paper compares these two algorithms to illustrate the time advantage of the divide and conquer strategy.

According to Figure 7, as the network scale increased, the running time of the BLMKM algorithm was very stable, while the running time of the dynamic programming algorithm and the HC algorithm grew exponentially. For Mybnet2 and Alarm networks, the dynamic programming algorithm did not run within the time (1 day) we specified, so we only show the trend here. This is because the dynamic programming algorithm hierarchically recurses the problem and has a high space occupation. It is difficult to obtain results for networks with more than 23 nodes. For the Alarm network, the HC algorithm takes about 15 h. The HC algorithm spends the most time calculating the maximum likelihood estimation and score of the candidate model, and finally obtains the approximate structure, so the time is relatively faster. The BLMKM algorithm proposed in this paper learns networks with the divide and conquer strategy and controls the time of exponential growth in a reasonable range. The idea of a block learning network can greatly reduce the time complexity. Compared with non-blocking classical structure learning algorithms, the BLMKM algorithm has a great time advantage.

**Figure 8:**
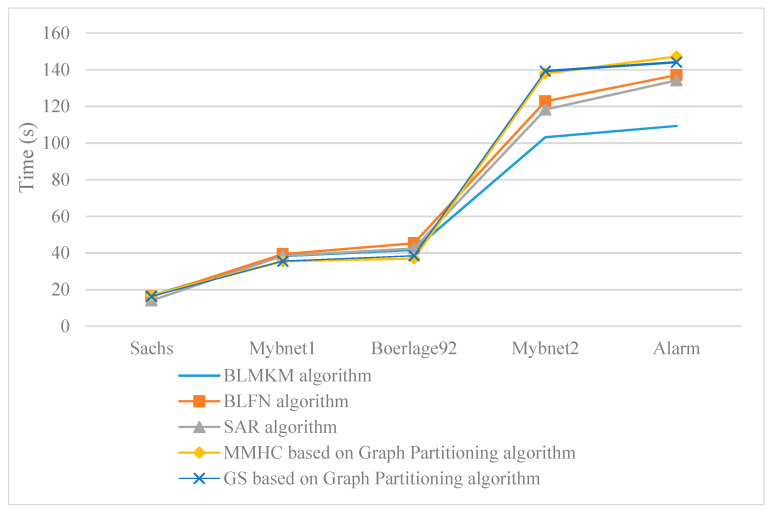
The comparison of running time between BLMKM algorithm and other block learning algorithms.

With the same sample size of 5000, we compared the BLMKM algorithm with the existing block learning algorithms in running time, as shown in Figure 8. The BLFN algorithm uses the classic community mining algorithm and the FN algorithm instead of the MKM algorithm to block the network; the SAR algorithm [15] is a new separation and merge algorithm proposed by H Liu et al. in 2017; the graph partitioning algorithm [14] is a graph segmentation method proposed by S Li et al. in 2014, including MMHC based on graph partitioning and GS based on graph partitioning. The SAR algorithm and graph partitioning algorithm are mentioned above. These four algorithms all adopt the idea of block learning, so we compared the BLMKM algorithm with them.

According to Figure 8, the time complexity of the five algorithms is not high. For larger Alarm networks, the running time of the five algorithms is less than 160 s, which shows that block learning network is beneficial to reducing the running time. The MKM algorithm can obtain the same accuracy as the FN algorithm, but the MKM algorithm consumes less time than the FN algorithm. It can also be seen from Figure 8 that the BLMKM algorithm runs faster than the BLFN algorithm. This also shows that the time performance of the block algorithm greatly affects the efficiency of the subsequent block learning algorithm, which verifies the necessity of improving the K-means algorithm in this paper.

For the Sachs, Mybnet1, and Boerlage92 networks, the running time of the five algorithms is not much different. This is because, for medium- to small-scale networks, the idea of block learning can reduce the time consumption to a certain extent, but due to the limitation of scale, the reduction effect is not obvious. For Mybnet2 and Alarm networks, the BLMKM algorithm is the fastest. This is because the BLMKM algorithm uses the dynamic programming algorithm to learn the structure, although the dynamic programming algorithm has the disadvantage of dimensional explosion, the learning time becomes small after blocking to reduce the dimensions. The dynamic programming algorithm learns the network hierarchically and recursively, which is very efficient. In this paper, the MMPC algorithm is used to prune branches, so the time consumption of the entire algorithm is not high. The SAR algorithm uses a learning algorithm based on scoring function, which is not constrained by the search space, increasing the number of candidate models that need to be measured, and the speed becomes slower after the network increases; MMHC based on graph partitioning algorithm and GS based on graph partitioning algorithm experience the problem of low efficiency when there are many variables, so it shows a disadvantage in running time when learning the Alarm network. For each small block, these two algorithms can meet the situation with few variables, so the time difference here is not large. The BLMKM algorithm has a certain time advantage over existing block learning algorithms.

#### 4.1.2. Comparison of Algorithm’s Accuracy

We analyzed whether the BLMKM algorithm can achieve the same accuracy as other algorithms, and the results are shown below. First, we used hamming distance for comparison, $A(N_{G}^{*})$ represents the added edges of the final learned network compared to the real network, $M(N_{G}^{*})$ represents the missing edges of the final learned network compared to the real network, $I(N_{G}^{*})$ represents the inverted edges of the final learned network compared to the real network. Hamming distance $H(N_{G}^{*})$ = $A(N_{G}^{*})$ + $M(N_{G}^{*})$ + $I(N_{G}^{*})$. The smaller the hamming distance between the final learned network and the real network, the higher the similarity between the final learned network and the real network, the higher the accuracy of the final learned network. The hamming distance is shown in Table 2.

For Sachs network, the SAR algorithm learns the network with the smallest hamming distance. The accuracy of the BLMKM algorithm proposed in this paper is mid-range. The learning of small networks belongs to the category that can be solved by non-blocking classical structure learning algorithms; the learning accuracy of the dynamic programming algorithm and HC algorithm learning is better. For other networks, the BLMKM algorithm has the highest accuracy. At this time, it shows the advantages of exact learning compared to the learning algorithm based on scoring function and the learning algorithm based on constraints. The dynamic programming algorithm had no results for the Mybnet2 and Alarm network learning within a specified time (1 day), so it is indicated by “-” in the table.

No matter which network, the $A(N_{G}^{*})$ of BLMKM algorithm, BLFN algorithm, MMHC based on graph partitioning algorithm, and GS based on graph partitioning algorithm were all 0. This is because these four algorithms use the MMPC algorithm as the network skeleton and it is added to the network learning as a constraint in subsequent learning, thereby ensuring that when learning the parents of a node, other redundant nodes are not considered; it will not learn more redundant edges compared to the real network. The SAR algorithm and other non-blocking classical structure learning algorithms will learn redundant edges. According to the hamming distance, the BLMKM algorithm proposed in this paper can achieve the same accuracy as other algorithms and has greater potential when the network becomes bigger.

At the same time, we also compared the BIC score of the BLMKM algorithm with other algorithms, as Table 3.

The BIC score can measure the similarity between the final learned network and the real network. The BIC score is an approximation of the edge likelihood function on the premise of large samples. The BIC score of the model structure is recorded as $BIC(N_{G}^{*}|D)$, that is

(9) $$\log P(D|N_{G}^{*})\approx \log P(D|N_{G}^{*},\theta^{*})-\frac{d}{2}\log m$$

The first term on the right is the optimal parameter logarithmic likelihood degree of the model $N_{G}^{*}$, which measures the fitting degree between structure $N_{G}^{*}$ and data D. The second term is a penalty term about model complexity, d is the number of independent parameters of the parameter vector θ, m is the sample size, and this penalty term makes the BIC score effectively avoid overfitting. The BIC score selects the model that fits the data and is relatively simple.

According to the above table, the data of the dynamic programming algorithm on the Mybnet2 and Alarm networks is missing because the learning of these two networks did not get results within the specified time (1 day). For the Sachs, Mybnet1, and Boerlage92 network, the BIC scores of the six algorithms were equivalent, but the hamming distance obtained by the six algorithms were different. This is because the BIC score is higher for the simpler model, but the hamming distance intuitively measures the difference between the final learned network and the real network edges. Both measure the accuracy of the algorithm from different points of view. For the Alarm network, the BLMKM algorithm proposed in this paper has the highest BIC score and the HC algorithm has the lowest BIC score, and the result of the hamming distance is consistent. This is because the HC algorithm easily falls into a local optimum, which leads to low accuracy and BIC score, but the BLMKM algorithm considers the final global network from the global scope to ensure the global optimality.

Summarizing the above experiments, it can be concluded that the BLMKM algorithm has a significant time advantage over the non-blocking classical structure learning algorithm, which can greatly reduce the time complexity. Compared with the existing block learning algorithms, the BLMKM algorithm has a certain time advantage. For example, the time consumption on the Alarm network is reduced by approximately 25%. By comparing the hamming distance and the BIC score, the BLMKM algorithm achieves the same accuracy as the current existing algorithms, has a slight improvement, and guarantees that the final learned network is globally optimal. The BLMKM algorithm greatly improves the learning speed under the premise of ensuring accuracy. For the Alarm network where the classical dynamic programming algorithm cannot obtain the learning results in a reasonable time, the BLMKM algorithm can quickly learn the global optimal network. The BLMKM algorithm is a feasible algorithm for learning the optimal BN for high-dimensional sparse data.

### 4.2. Experiments for Analysis of Radar Effect Mechanism

Radar first appeared on the battlefield of World War I and was an effective means for the British to detect German aircraft. After several decades of development, radar has become one of the most commonly used ways to detect aircraft, in all detection methods, not only in military use but also in the civilian field [29]. Nowadays, a variety of radars can detect sea, land, or air objects, and can also calculate the moving direction and even speed to help us predict the movement of objects. However, with the widespread application of radar, it has also been found that the electromagnetic waves emitted at certain frequencies can interfere with the radar detection results, especially when the interference power is large enough, radar can be completely disabled [21,30]. Later, people researched various radar interference methods, such as signal suppression and false target deception. There are some inherent electromagnetic noises in the environment, such as naturally generated electromagnetic waves and electromagnetic waves of other communication equipment. These unrelated electromagnetic waves will also affect the normal use of radar [31]. Thus, the detection accuracy of the radar will be reduced due to the presence of these interferences in a complex electromagnetic environment, how to select the relevant parameters of the radar to reduce the impacts on the detection accuracy, and which parameters have a greater influence on the final detection accuracy become an important research issue.

This issue can be considered as the analysis of the radar effect mechanism. In the field of complex electromagnetic effects, some factors can cause certain specific effects to occur. When an effect occurs, a set of inverse problem research methods from effect to factors can be established. In many situations, we do not know which factor is interfered. Through setting the effect inversely, the combination of a series of factors can be obtained to realize the inverse inference process and then find the causes of interference.

At present, the analysis of the radar effect mechanism mostly adopts the traditional method-equations calculation. Due to a large number of variables, the calculation is complicated and slow, the qualitative and quantitative relationship between “effects-factors” cannot be integrated as a whole, and the state of the input cannot be inferred backward from the artificially obtained output. To address this issue, BN is introduced to analyze the radar effect mechanism. Compared with the traditional method, BN can contribute to the model of the radar effect mechanism faster. Each node in BN represents a variable and the model can contain all variables in radar effect mechanism. It can also change the structure when the quantity and variety of variables change. After the model is established, all variables can be inquired directly, which is much more convenient and intuitive than the traditional method. BN can not only reason efficiently, but also infer backward, and with well-trained BN, we can guide the parameter optimization and even predict the detecting result.

**Figure 9:**
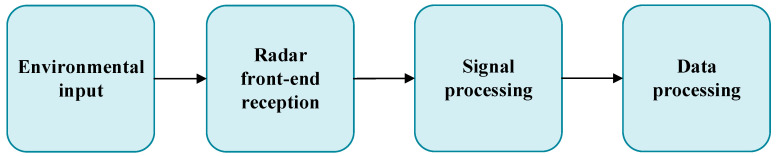
The four stages of the analysis of radar effect mechanism.

This paper uses actual measurement data for analysis. The data of radar effect mechanism is measured from the characteristic parameters which can represent the effect phenomena or effect factors of each key links. Several parameters are settled and change others. These four key links are environmental input, radar front-end reception, signal processing, and data processing, which are shown in Figure 9. Then research the mechanism of the “effect-factor” problem of these four key links, that is, the relationship between each key link and the detection effect.

According to the type of signal received by the radar, add the corresponding interference signal and record the relevant parameters of the radar subsystems and the final error of the target detection under certain interference. In this process, the parameters of each key link need to be analyzed simultaneously, and the adjustment range of optional parameters is relatively large, therefore, the research in this paper is based on a fixed waveform to interfere with the radar system. Using BN, we can predict the detecting result with different interference factors and guide parameter optimization by setting most of the parameters of the radar system.

The recorded data of the radar system is composed of four categories according to the key links: environmental input signal parameters, radar front-end reception parameters, signal processing (back-end) parameters, and data processing parameters. The original data has 42 parameters that belong to high-dimensional data and can be regarded as sparse data for these four categories. Due to too many parameters, the parameters of these four categories are listed as Table 4.

There are many factors that will affect the detection accuracy, like waveform, signal processing, data processing strategy, and so on. To analyze the effect of interference and simplify the problem, other parameters such as clutter Doppler spectral width and improving factors are fixed.

By abandoning these steeled parameters to clear up the data, we get eight parameters in environmental input, three parameters in radar front-end reception, 11 parameters in signal processing, and four parameters in data processing, so each input data is 26-dimensional.

This high-dimensional pure numerical data is more complicated for the traditional equation calculation, and it takes more manpower and material resources. Once one step is wrong, the subsequent calculation is also wrong. At this time, the BN model can be used as a new model to describe and analyze the radar effect mechanism, but the traditional BN learning algorithm is difficult to compromise between modeling accuracy and speed for high-dimensional data. For BN modeling of this high-dimensional data, the BLMKM algorithm proposed in this paper can improve the running speed while ensuring accuracy. Therefore, the BLMKM algorithm can be used to design the corresponding BN model according to the actual situation of radar effect mechanism analysis. The BN model can analyze all the relationships of all these variables simultaneously in radar effect mechanism data. To simplify the analysis and obviously indicate the effect mechanism of radar, this paper settles the detection errors (point trace error and track error) as the outputs. The following analysis will all be based on this assumption.

#### 4.2.1. Construct BN Model—The BLMKM Algorithm

First, divide the state of the filtered data. Because the distribution range of the error data is relatively large, some errors are much larger than most other error values. Therefore, the larger error is regarded as a singular value, and the state is divided separately. The densely distributed error values are divided into states according to dense intervals, which can divide the number of data states more finely, making the analysis of the data more detailed.

After the state division is completed, these data are used as input and the BN model is learned through the BLMKM algorithm and the causal relationship between each variable can be obtained. The average running time of the BLMKM algorithm is 112.37 s. The constructed BN model of the radar effect mechanism is as follows.

**Figure 10:**
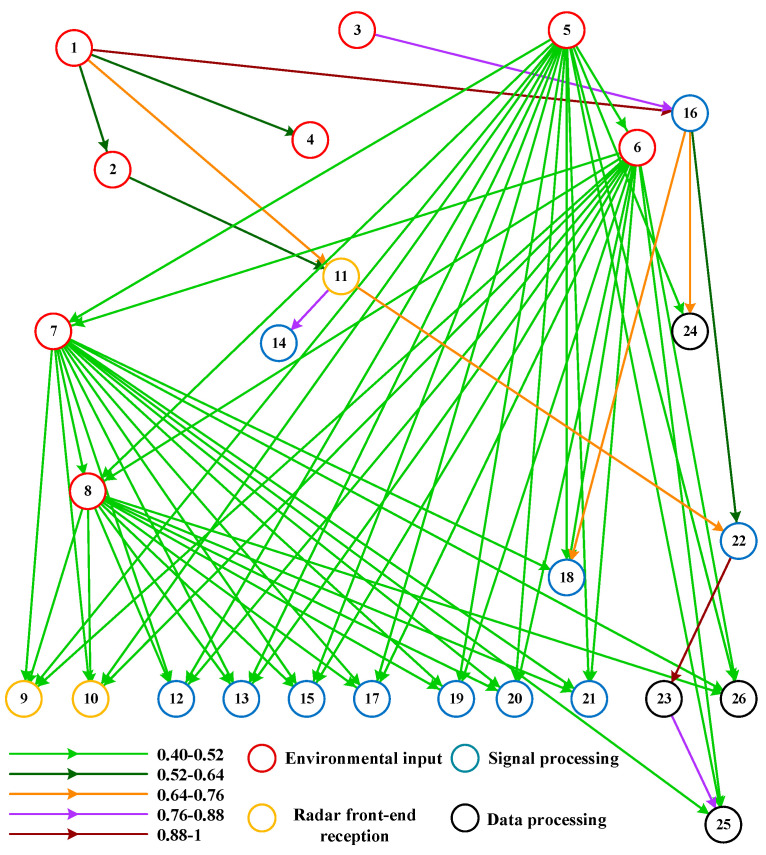
The BN model of radar effect mechanism.

The nodes in Figure 10 represent the parameters of these four key links, and the directed edges represent the relationships of these parameters. The strength of the relationship is represented by different colors. The final error is indicated by node 23 and node 25, and there are fewer nodes connected to these two error nodes. Node 23 is connected to node 22, while node 22 is connected to node 11 and node 16, these two nodes (11 and 16) belong to the signal processing subsystem. Node 23 is directly connected to node 25. Overall, the network model constructed by the BLMKM algorithm conforms to the actual situation and logical structure. Compared with the traditional equation calculation method and computer simulation, the constructed BN model can intuitively and clearly describe the relationship between the variables in the radar system from qualitative and quantitative perspectives and can also complete the inverse problem of the radar effect mechanism faster. Therefore, it can be used as a new method for analysis of the radar effect mechanism.

#### 4.2.2. Inverse Problem Analysis—BN Inference

After that, according to the above BN model, complete the inverse problem analysis of the radar effect mechanism by BN inference. The junction tree algorithm [16] is used to accurately infer the states of the radar variables when the states of errors are given.

**Figure 11:**
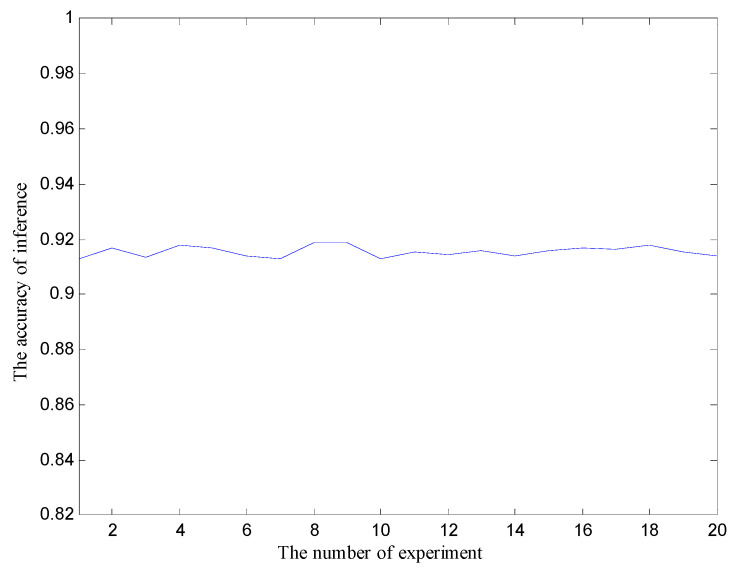
The accuracy of inference based on the BLMKM algorithm modeling.

After the test of 7962 sets of test data, the average inference time is 520.76 s. The model built by the BLMKM algorithm has a faster inference speed than the traditional equation calculation method. The average inference accuracy of the model is about 91.56%, as shown in Figure 11. That is, the reverse inference accuracy of the radar effect mechanism is about 91.56%, indicating that the constructed BN model tends to be more realistic and can accurately describe the relationship between the errors and other variables in the radar system. As a block exact learning algorithm, the BLMKM algorithm proposed in this paper improves the learning speed while still maintaining the modeling accuracy.

In the analysis of the radar effect mechanism, the accuracy of point trace error and track error obtained by inference corresponds to the accuracy of the radar detection target. According to the error, we can judge whether the detection results given by the radar are worth adopting. In a complex actual environment, the overall detection accuracy of the radar system can be improved by eliminating high-error results in radar detection data and retaining accurate results. At the same time, in the process of detecting or locking a single target, the radar detection results are analyzed by using the radar effect mechanism analysis method to determine whether the radar detects the target accurately, which can provide a reference for the final decision. When some variables like interference parameters are known, we can also set the rest parameters like the waveform we used in our radar system to be inversed to help us to select the appropriate parameters.

The experiments in this section show that under certain interference conditions, the parameters of the signal processing subsystem of the radar system have a greater influence on the final detection error. When analyzing the radar effect mechanism, the BLMKM algorithm proposed in this paper can obtain BN model of radar effect mechanism faster with high accuracy and complete the inverse problem analysis by BN inference. BN can also help predict the detecting result and guide the parameter optimization. By using the BLMKM algorithm, we can model other high-dimensional and sparse data, which has certain practical value and potential application prospects.

## 5. Conclusions

By improving the conventional K-means algorithm and combining it with dynamic programming algorithm and other methods, this paper proposes the BLMKM algorithm to learn the accurate structure of Bayesian networks, especially for high-dimensional and sparse data. The improved K-means algorithm (MKM algorithm) can block the nodes of the Bayesian network more reasonably and obtain the precise structure of the network through the dynamic programming algorithm (MMPC) and other methods. Experiments show that the BLMKM algorithm can obtain an accurate structure of a network with the same accuracy as the classic non-blocking learning algorithms in a shorter time, especially when learning high-dimensional and sparse networks. To verify the ability to deal with actual high-dimensional and sparse problems, BLMKM is used to learn radar effect mechanism dataset. Compared with the traditional analysis method, the BLMKM algorithm can obtain higher accuracy quickly. With the ability of BN inference, the radar effect mechanism dataset can be further analyzed. It can be seen that BLMKM is an efficient accurate structure learning algorithm for modeling high-dimensional and sparse data.

Although the proposed algorithm in this paper has achieved good results in many aspects, it still needs further improvement. For example, for the analysis of the radar effect mechanism, how to obtain inference results more accurately will be the direction of our subsequent research.

## Figures and Tables

**Table 1:** The parameters of networks.

The Name of Networks	The Number of Nodes	The Number of Edges
Sachs	11	17
Mybnet1	18	27
Boerlage92	23	36
Mybnet2	32	42
Alarm	37	46

**Table 2:** The hamming distance of seven algorithms.

	BLMKM Algorithm	BLFN Algorithm	SAR Algorithm	MMHC Based on Graph Partitioning Algorithm	GS Based on Graph Partitioning Algorithm	Dynamic Programming Algorithm	HC Algorithm
Sachs							
$A(N_{G}^{*})$	0	0	0	0	0	1	1
$M(N_{G}^{*})$	3	3	0	3	3	0	0
$I(N_{G}^{*})$	4	3	2	6	6	3	4
$H(N_{G}^{*})$	7	6	2	9	9	4	5
Mybnet1							
$A(N_{G}^{*})$	0	0	0	0	0	3	4
$M(N_{G}^{*})$	2	3	1	2	2	2	2
$I(N_{G}^{*})$	6	8	3	6	11	12	6
$H(N_{G}^{*})$	8	11	4	8	13	17	12
Boerlage92							
$A(N_{G}^{*})$	0	0	3	0	0	7	0
$M(N_{G}^{*})$	9	9	6	10	9	3	10
$I(N_{G}^{*})$	5	7	7	8	8	8	9
$H(N_{G}^{*})$	14	16	16	18	17	18	19
Mybnet2							
$A(N_{G}^{*})$	0	0	1	0	0	-	0
$M(N_{G}^{*})$	5	6	5	7	7	-	8
$I(N_{G}^{*})$	9	9	9	11	9	-	6
$H(N_{G}^{*})$	14	15	15	18	16	-	14
Alarm							
$A(N_{G}^{*})$	0	0	7	0	0	-	8
$M(N_{G}^{*})$	9	11	4	8	11	-	5
$I(N_{G}^{*})$	6	8	4	14	9	-	12
$H(N_{G}^{*})$	15	19	15	22	20	-	25

**Table 3:** The BIC score of six algorithms.

	BLMKM Algorithm	BLFN Algorithm	MMHC Based on Graph Partitioning Algorithm	GS Based on Graph Partitioning Algorithm	Dynamic Programming Algorithm	HC Algorithm
Sachs	−3.7004	−3.7907	−3.7038	−3.7813	−3.6616	−3.6753
Mybnet1	−5.0766	−5.0823	−5.0833	−5.0853	−5.0946	−5.0773
Boerlage92	−5.0474	−5.0754	−5.0853	−5.0773	−5.0650	−5.0758
Mybnet2	−9.1036	−9.1300	−9.1478	−9.1459	-	−9.0947
Alarm	−6.5210	−6.7179	−6.7889	−6.7486	-	−6.8439

**Table 4:** Some parameters of radar effect mechanism data.

Data Type	Parameter Type
Environmental input signal parameters	Suppress interference power (dBm)
	Suppress interference bandwidth (MHz)
	Spoofing interferes with the false target interval (μs), etc.
Radar front-end reception parameters	Spectrum average (dB), etc.
Signal processing(back-end) parameters	Digital down-conversion spectrum peak (dB)
	Spectrum average (dB)
	3dB bandwidth (MHz), etc.
Data processing parameters	Point trace error (m), etc.
